# Commentary: Effects of ALS-associated TANK binding kinase 1 mutations on protein-protein interactions and kinase activity

**DOI:** 10.3389/fnins.2020.551006

**Published:** 2020-09-11

**Authors:** Axel Freischmidt, David Brenner, Albert C. Ludolph, Peter M. Andersen, Jochen H. Weishaupt

**Affiliations:** ^1^Department of Neurology, Ulm University, Ulm, Germany; ^2^German Center for Neurodegenerative Diseases (DZNE) Ulm, Ulm, Germany; ^3^Division for Neurodegenerative Diseases, Neurology Department, University Medicine Mannheim, Heidelberg University, Mannheim, Germany; ^4^Department of Clinical Science, Neurosciences, Umeå University, Umeå, Sweden

**Keywords:** amyotrophic lateral sclerosis, ALS, frontotemporal dementia, FTD, TBK1, neuroinflammation, genetic counseling, genetic variants

## The Vast Majority of *TBK1* Missense Variants Are of Unknown Pathogenicity

TANK-binding kinase 1 (TBK1) is a serine/threonine kinase involved in the regulation of essential cellular functions, including innate immunity and selective autophagy (Helgason et al., 2013). Loss-of-function mutations have been demonstrated to cause amyotrophic lateral sclerosis (ALS) and frontotemporal dementia (FTD) (Cirulli et al., 2015; Freischmidt et al., 2015). Recently, Ye et al. (2019) reported functional effects of 25 *TBK1* missense variants found in patients with ALS or FTD. They focus on impaired protein-protein interactions and substrate-specific defects in pathways such as innate immunity and autophagy. The authors describe that “TBK1 ALS mutations display a variety of defects” in different cellular pathways and conclude that “multiple defects can be caused by mutations from a single disease-associated gene but can lead to the common pathogenic outcome.”

We would like to point out that the genetic link between *TBK1* and neurodegeneration is largely based on Mendelian dominantly inherited deleterious loss-of-function mutations (such as splice site or frameshift mutations), which are enriched in ALS/FTD patients and result in a loss of expression of one *TBK1* allele (Freischmidt et al., 2015). Importantly, several splice-site variants of *TBK1* (Freischmidt et al., 2015) as well as an in-frame deletion of a single amino acid [TBK1 ΔE643; (Gijselinck et al., 2015)] have been shown to co-segregate with the disease over multiple generations.

In stark contrast, rare or unique *TBK1* missense variants are also frequently detected in healthy controls (Cirulli et al., 2015; Freischmidt et al., 2015), although, overall, being significantly enriched in ALS/FTD patients compared to controls (Cirulli et al., 2015). However, the study by Cirulli et al. (2015) does not allow drawing conclusions about the pathogenicity of any specific *TBK1* missense variant. Moreover, little or no data about co-segregation with disease are available with regard to the vast majority of these missense variants. The fact that an extremely rare or novel variant was detected in single or very few unrelated individuals [see e.g., (Freischmidt et al., 2017)] with a certain disease is by far not a proof of causality. Significant co-segregation with the disease (preferably in more than one family) or enrichment of the single, specific variant in patients compared to controls would provide a more solid body of evidence for causality of a specific variant. Hence, in our view (Freischmidt et al., 2017) and in accordance with the ACMG Standards and guidelines for classifying pathogenic variants (Richards et al., 2015) almost all missense variants in *TBK1* must be considered “variants of unknown significance” (VUS). While a certain percentage of these *TBK1* missense variants may of course be pathogenic, it is highly likely that some or even most of them represent weak risk factors, or are unrelated to ALS/FTD causation.

Therefore, referring to *TBK1* missense variants detected in ALS patients as (pathogenic) “ALS mutations” (Ye et al., 2019) is misleading. For the unclear causality, a correlation between different distinct downstream effects of *TBK1* variants and ALS/FTD is currently impossible.

Consequently, while the value of the study by Ye et al. (2019) for the structural biological understanding of TBK1 function is undisputable, their conclusion that impairment of various different cell biological TBK1 functions may result in ALS/FTD is based on analyses of “variants of unknown significance” and is currently not supported by genetic evidence. We think that this should be taken into careful consideration when interpreting experimental results based on *TBK1* missense variants, but also with regard to the genetic counseling of affected individuals and their families.

## Author Contributions

All authors listed have made a substantial, direct and intellectual contribution to the work, and approved it for publication.

## Conflict of Interest

The authors declare that the research was conducted in the absence of any commercial or financial relationships that could be construed as a potential conflict of interest. The handling editor declared a past co-authorship with one of the authors PA.
